# Medical Statistics

**Published:** 1838-01

**Authors:** 


### MEDICAL STATISTICS.

*Statistics of the Sickness and Mortality which occurred among the Troops employed on the Expedition to the Scheldt, in the year* 1809. By Henry Marshall, Deputy Inspector-General of Army Hospitals.

This is a most valuable contribution to medical statistics, and proves how admirably fitted its author is for the task in which we are happy to learn from this paper that he is engaged, along with Lieut. Tulloch, viz. "the compilation of the statistics of the British Army, for the purpose of ascertaining the extent of the sickness and mortality of the troops, together with the prevailing diseases among the men in each military command." We are gratified to learn from the same authority, that some of these reports are already finished, and are forthwith to be published.

The present paper gives a full report of the medical tragedy of the Walcheren expedition, and places in the most striking light, at once the immense value of a knowledge of medical topography and medical statistics, and of the utter ignorance of these by the projectors and directors of this ill-fated armament. We can only find room for a few extracts and remarks; but we recommend the paper earnestly to the attention of our readers.

*Medical Topography of Walcheren.* "The surface of the islands of Zealand is flat and low, being in some places below the level of the sea at high-water. As the water percolates through the banks, and accumulates by rains, much care and labour is required to remove it, which is commonly effected by means of sluices and mills. The water is collected in ditches and canals, and conveyed to the points where it can be most conveniently discharged over the banks. Inundations also occasionally occur, and as the water stagnates, the grounds are frequently left covered with slime and mud. The soil is excessively fertile, and produces corn, abundance of fruit, vegetables, and madder."

*Its Influence on Strangers.* "Dr. Wind, who translated into Dutch Dr. Lind's Essay on preserving the Health of Seamen, and who practised medicine in Walcheren for many years, informs us that the Scotch regiment in the Dutch service has been known to bury their whole numbers at Sluys, in Dutch Flanders, in three years. We learn from the report of Dr. Borland and his coadjutors, that, upon an examination of the sick returns of the French army for a period of seven years, it was found that at least one-third or 33 per cent. of its force was annually cut off by endemic disease. I may add, that when the English landed in Walcheren, there were only eighty-five men alive in a Dutch regiment, which at its arrival there, three years before, was eight hundred strong. The annual ratio of mortality in this corps must have been about 31 per cent., or rather more than double the mean ratio of mortality which occurs among troops in Jamaica."

Into this hot-bed of malaria were upwards of 60,000 men sent in the beginning of August; and here they remained until they were forced to reembark, a broken and disgraced host, vanquished by the elements, not the enemy. In the month immediately succeeding that of the debarkation, we find the proportion of the sick to the well exactly *one-half!* and the mean ratio of mortality, from the commencement of the expedition in July to the end of January following, (about six weeks after the reembarkation of the troops,) was among the officers 3.7, and among the men 10.3 per cent.! Six weeks after Walcheren was evacuated,—that is, after the return of the troops to England,—one-third of the troops of the line were on the sick-list, and of these a large proportion died. Nor did the evil terminate here. "Long after this date, (says Mr. Marshall,) many of the men who had apparently escaped the obnoxious influence of the climate of Walcheren were attacked, and suffered severely from the specific endemic disease, so that a very extended investigation would be required before a near approximation could be made of the consequences of the expedition to the Scheldt, in destroying the efficiency, and promoting the mortality of the British army. It is well known that among the regiments which had been employed in Walcheren, and which served afterwards in the Peninsula, many of the men were, upon the first exposure to cold and fatigue, rendered unfit for duty, so as frequently not to leave one-third of the strength fit for service."

After reading these statements every one must cordially assent to Mr. Marshall's proposition, that "the topography and statistics of the different colonies and dependencies to which troops are sent should form part of the education and study of every military man." Most certainly they ought to be studied by every medical man who enters into the public service; and we would here suggest to the present enlightened directors of our naval and military medical establishments the expediency of enabling every medical officer, before entering on active duty, to profit by the attendance on a course of lectures specially dedicated to this and other subjects of naval and military Hygiène. If, for this purpose, a special professorship were necessary,—and here both branches of the public service might be combined, —we are sure the public funds could not be more beneficially appropriated than in its establishment: and we doubt if the proposed office could be better filled than by the enlightened author of the paper now before us.

*Edinburgh Journal. October,* 1837.

---

### *Of the Probability of Death and Recovery in Asiatic Cholera.* By WM. FARR, Esq.

[THE following extract is from a very valuable paper *on Prognosis*, which, like all Mr. Farr's productions, exhibits a profound acquaintance with the more recondite and philosophical parts of Medical Statistics. Our limits prevent us from laying more of this article before our readers; but we regret this the less, as we presume the small work in which it appears will be seen by most of them. We can conscientiously recommend the British Medical Almanack to their notice, as containing an infinity of matter of the greatest interest and importance; and we consider the profession under great obligations to Mr. Farr for the manner in which he continues to conduct it.]

*Table of* 4907 *fatal Cases of Cholera, shewing the Number remaining alive at each of* 16 *Periods; and the Number dying in the Period following.*

| Hour. | To die. | Dying.* | Day. | To die. | Dying. | Day. | To die. | Dying. |
|---|---|---|---|---|---|---|---|---|
| 0 | 4907 | 204 | 1 | 2523 | 823 | 7 | 372 | 171 |
| 6 | 4703 | 615 | 2 | 1700 | 502 | 8 | 201 | 35 |
| 12 | 4088 | 392 | 3 | 1198 | 382 | 9 | 166 | 36 |
| 18 | 3696 | 1173 | 4 | 816 | 240 | 10 | 130 | 111 |
| | | | 5 | 576 | 125 | 15 | 19 | 19 |
| | | | 6 | 451 | 79 | 20 | 0 | |

The tendency to speak in weeks, and well-known periods, produced the irregularity at the 7th day. Of the 171 thrown on that day, some died a day or two before, some a day or two afterwards. For the same reason it may be safely admitted that the deaths increased regularly on the first day. The daily rate of mortality in the first 12 hours was 16 per cent.; in the next 12 hours (12—24) 37 per cent.; in the 2d day, 11 per cent.; in the 3d day, 8 per cent., if the mortality of cases of cholera in Paris was 49 per cent.—it could not have been higher,—and none of the severe cases were cured in the first 3 days. The force of mortality attained its maximum in cholera by the 21st hour (18—24 hours); the maximum intensity in small-pox is attained in days 10—15; in phthisis, in 6—9 months. Taking a year as the unity of time, the relative maximum force of mortality—the deaths out of 100 constantly living,—in the height of these three diseases is, Cholera, 13614; Small-pox, 1150; Phthisis, 148. The danger of cholera decreases as the time advances; the longer a cholera patient lives, the more likely he is to live. The way in which the prognosis becomes favorable is shewn in the following table:

*Table of the Probability of Recovery from the severer Attacks of Cholera at the end of* 12 *hours, and* 1, 2, *and* 3 *days.*

| Cases. | | ToRecover. | To Die. | Probability of Recovery. | |
|---|---|---|---|---|---|
| 0 hours | 10000 | 5093 | 4907 | .509 nearly | 1 to 1 |
| 12 ...... | 9181 | 5093 | 4088 | .555 ...... | 1.3 ... 1 |
| 1 day | 7616 | 5093 | 2523 | .669 ...... | 2 ... 1 |
| 2 ...... | 6793 | 5093 | 1700 | .750 ...... | 3 ... 1 |
| 3 ...... | 6291 | 5093 | 1198 | .809 ...... | 4 ... 1 |

These facts prove that, in cholera, the probability is generally not in favour of death; they also establish the importance of early treatment, for half the deaths happen in the 24 hours. What the practitioner does, he should do quickly.

*British Medical Almanack for* 1838.

* Rapport sur la Marche et les Effets du Cholera Morbus dans Paris et le department de la Seine.

